# Implicit learning of predictable sound sequences modulates human brain responses at different levels of the auditory hierarchy

**DOI:** 10.3389/fnhum.2015.00505

**Published:** 2015-09-16

**Authors:** Françoise Lecaignard, Olivier Bertrand, Gérard Gimenez, Jérémie Mattout, Anne Caclin

**Affiliations:** ^1^Lyon Neuroscience Research Center, CRNL, INSERM, U1028 – CNRS, UMR5292, Brain Dynamics and Cognition TeamLyon, France; ^2^University Lyon 1Lyon, France; ^3^MEG Department, CERMEP Imaging CenterLyon, France

**Keywords:** mismatch negativity, auditory regularity, predictive coding, early deviance response, EEG, P3a

## Abstract

*Deviant* stimuli, violating regularities in a sensory environment, elicit the mismatch negativity (MMN), largely described in the Event-Related Potential literature. While it is widely accepted that the MMN reflects more than basic change detection, a comprehensive description of mental processes modulating this response is still lacking. Within the framework of predictive coding, deviance processing is part of an inference process where prediction errors (the mismatch between incoming sensations and predictions established through experience) are minimized. In this view, the MMN is a measure of prediction error, which yields specific expectations regarding its modulations by various experimental factors. In particular, it predicts that the MMN should decrease as the occurrence of a deviance becomes more predictable. We conducted a passive oddball EEG study and manipulated the predictability of sound sequences by means of different temporal structures. Importantly, our design allows comparing mismatch responses elicited by predictable and unpredictable violations of a simple repetition rule and therefore departs from previous studies that investigate violations of different time-scale regularities. We observed a decrease of the MMN with predictability and interestingly, a similar effect at earlier latencies, within 70 ms after deviance onset. Following these pre-attentive responses, a reduced P3a was measured in the case of predictable deviants. We conclude that early and late deviance responses reflect prediction errors, triggering belief updating within the auditory hierarchy. Beside, in this passive study, such perceptual inference appears to be modulated by higher-level implicit learning of sequence statistical structures. Our findings argue for a hierarchical model of auditory processing where predictive coding enables implicit extraction of environmental regularities.

## Introduction

Oddball paradigms involve sequences of a repeating (standard) pattern that sets up a regular environment, and infrequent (deviant) stimuli, which violate this regularity and subsequently elicit mismatch responses in the brain. They have been extensively employed in humans using non-invasive electrophysiology recordings, because of their ease of recording, their unique ability to reveal mechanisms of perceptual inference and learning (Kujala and Näätänen, 2010), as well as their clinical relevance (Näätänen et al., 2012; Morlet and Fischer, 2014). The well-known mismatch negativity (MMN), first described in Näätänen et al. (1978), is observed in such paradigms and has been described in several sensory modalities although mostly studied in audition (for review, see Näätänen et al., 2007). A large literature is dedicated to the functional interpretation of the MMN and several models, resting either on psychological concepts, on computational frameworks or even on neural adaptation processes have been proposed [for review, see Näätänen et al. (2007) and Garrido et al. (2009b)]. Adaptation refers to a decrease of neural responsiveness after several repetitions of a stimulus, and is widely acknowledged to contribute to the difference in responses to standards and deviants. A considerable number of MMN findings argue against the adaptation model (that implies a full account of the MMN by adaptation effects) and suggest that this component reflects an automatic detection of change in the acoustic environment, with strong support to the MMN as the output of a comparator between observed and expected sensory inputs (Näätänen et al., 2007). In the current study, we were interested in recent theories based on a predictive coding scheme that have been proposed to account for the generation of the MMN (Friston, 2005) [see also Winkler and Czigler (2012) for a review of findings compatible with this account]. These theories rest upon a hierarchical organization of the brain, wherein predictions regarding incoming inputs are conveyed to lower levels by top-down messages, while bottom-up prediction errors reflecting mismatch between observations and predictions are sent back to higher levels. In this view, the MMN reflects a prediction error that triggers the update of predictions by means of message-passing between the different levels of the auditory hierarchy (Friston, 2005).

Importantly, predictive coding models of mismatch responses do not entail a single prediction regarding incoming inputs but multiple ones, generated at different levels of the hierarchy (Friston, 2005). Precisely, these predictions pertain to the physical attributes of sound and to the statistical dependencies within the sound sequence. Accordingly, prediction errors, hence likely the MMN, should be affected by at least three factors: (1) the acoustic separation between the predicted and observed stimuli (also referred to as the deviance magnitude), (2) the variability of the acoustic features, and (3) the sequence predictability, deriving from statistical regularities. Factor (1), deviance magnitude, has already been proved to modulate the MMN. For instance, Tiitinen et al. (1994) showed that for frequency deviation spanning above 2% of the standard frequency, the larger the deviation, the larger the MMN amplitude. The last two factors affect prediction error through modulations of sound predictability, by influencing either the predictability of the sound’s acoustic features [factor (2)], or the predictability of the stimulus category [standard or deviant, factor (3)]. Importantly, predictability may influence both the content of the prediction and its precision or confidence. The two evolve with learning and could modulate the MMN amplitude, provided that the MMN reflects a precision-weighted prediction error (Friston and Kiebel, 2009). Consequently, we hypothesized that the MMN amplitude should be reduced as the occurrence of the deviant stimulus becomes more predictable.

In the two following sections, we review the findings describing effects of above-defined factors 2 and 3 on the MMN amplitude. It reveals that they have been rarely studied so far, probably because of the methodological difficulties to disentangle those effects from those of deviance magnitude. Yet, validating the above hypothesis is required in order to assess the predictive coding perspective on the MMN and to refine our functional understanding of this widely used electrophysiological marker. The present study was carefully designed to overcome methodological caveats and specifically observe the effect of sequence predictability on the MMN.

### Effect of Acoustic Feature Variability on the MMN

Among the few studies that investigated the effect of predictability on the MMN, the majority manipulated the variability of the acoustic features of standard stimuli. In Daikhin and Ahissar (2012), the authors used a frequency oddball sequence with variable standard frequency belonging to a uniform distribution with a 2% deviation. Compared to a fixed standard condition, the authors found no significant difference in average responses to standards but a reduced MMN. This suggests that conditions with jittered standards yield a blurred representation of the standard stimulus, producing a less precise prediction and hence weaker responses to deviance. More recently, larger deviations were used (Garrido et al., 2013), with sequences of sounds whose frequencies were drawn from either a narrow or a broad Gaussian distribution (mean frequency of 500 Hz with standard deviations of 250 and 1500 Hz, respectively). Outlier sounds elicited an MMN-like response, which was reduced in the case of the broad distribution. This confirms the ability of the brain to extract statistical rules from sound sequences and gives strong support to the existence of predictions of future events that would be weighted by their inferred precision.

However, since these studies manipulated the predictability of the standards in ways that inherently involve changes in the acoustic parameters, the observed results might be confounded with deviance magnitude and adaptation effects (induced by refractoriness) that are likely to differ between conditions.

### Effect of Sequence Predictability on the MMN

Sequence (or sound category) predictability refers to rules that define the statistical dependencies of items within the sequence. Rules are usually categorized into simple (local) ones resting on short time-scale dependencies and complex (abstract or global) ones generating larger time-scale regularities or contingent relations. The violation of the latter also elicits a MMN (in both cases of passive and active paradigms) and has largely been described in the literature (for review, see Näätänen et al., 2010). Passive studies used the MMN as a marker of rule violation in order to reveal fairly high-level implicit learning processes (see for instance Bendixen et al., 2008; Todd et al., 2013). They were, however, not designed to test the effect of sequence predictability on the MMN *per se*.

Deviant predictability should be distinguished from deviant probability. The latter refers to the ratio of deviant events within the sequence, irrespective of its temporal structure, while the former refers to the statistical nature of the temporal sequence, irrespective of deviant occurrence frequency. Some studies have manipulated the deviant probability in order to measure its effect on the MMN (Sams et al., 1983; Sato et al., 2000). In our study, we manipulated deviant predictability only, which avoids the confounding effect of refractoriness inherent to the manipulation of deviant probability (i.e., varying the number of standards preceding a deviant).

To date, only a couple of studies have compared MMN responses elicited by unpredictable sequences (embedding *unpredictable* deviants) and predictable ones (embedding *predictable* deviants). In Scherg et al. (1989), a fully predictable sequence (*one frequency deviant every fifth tone*) was compared with an unpredictable one with the same global deviant probability (*p* = 0.2). The authors found no significant effect of the predictability manipulation on the MMN amplitude. They hypothesized that this result was compatible with initial findings (and widely confirmed since) suggesting that the MMN derives from an automatic process independent of participant’s attention (Näätänen et al., 1978, 2010). However, using the same paradigm but with different temporal characteristics, Sussman et al. (1998) and Sussman and Gumenyuk (2005) found a disappearance of the MMN in the predictable condition, which the authors interpreted as an automatic perceptual effect of tone grouping that could only occur in the predictable condition. However, as judiciously pointed by Fishman (2014), this effect could also be attributable to predictability. Importantly though, none of these studies rigorously controlled for adaptation effects as the number of standard preceding a deviant differed between the regular and irregular conditions. Others studies proposed oddball sequences embedding predictable deviants (Jankowiak and Berti, 2007; Bekinschtein et al., 2009) but their aim was not to measure the effect of predictability on mismatch responses. In some respect, although using a very different setting, a few studies already reported MMN-like responses that were modulated by the predictability of musical sequences. For instance, in Brattico et al. (2006), out-of-key tone responses suggest that less probable transitions are processed like deviants. In Vuust et al. (2009), subtle rhythmic violations were shown to induce larger magnetic MMN-like responses in musical experts compared to novices, whereas large violations induced responses in both groups. In line with those studies, the current experiment aims at generalizing those findings by testing the effect of predictability in isolation of deviance magnitude and independently of acquired skills over the lifespan.

From the existing literature briefly reviewed here, it is clear that empirical findings are compatible with the predictive coding view of the MMN. Nevertheless, direct evidence is missing and finely controlled sequence predictability appears as a good candidate to resolve this issue. As reported above, little is known on the effect of sequence predictability on the MMN, since it has never been studied genuinely. The widely acknowledged automaticity of the MMN has possibly inclined to the worthlessness of searching for any predictability-driven modulation. Today, recent (computational) theories of brain function (Friston, 2005; Winkler and Czigler, 2012) rather suggest that sequence predictability should affect deviance responses as follows: the more predictable the occurrence of a deviant sound, the finer the prediction, hence the smaller the prediction error and the smaller the MMN amplitude. Therefore, we used a passive oddball paradigm with unpredictable and predictable sound sequences differing by the transitional probabilities between sounds within each sequence type. The strict conservation of the acoustic properties of the sequence between conditions was achieved by means of a statistical structure determined over a relatively long time range in the predictable condition. Our design also includes the appropriate control for adaptation effects. Furthermore, we used small deviance magnitudes in a passive oddball paradigm, in order to limit automatic attention-orienting processes. These processes are typically reflected by the N2b-P3a complex (brain orienting response) following the MMN under specific condition of attention (Näätänen et al., 1982; Morlet et al., 2014). As mentioned above, the ability of the brain to encode implicitly large time-scale regularities has been indirectly demonstrated in several MMN studies, therefore we expected that participants would learn the statistical rule in the predictable condition. We hypothesized that predictable deviants would elicit reduced deviance responses. Conversely, in the absence of any implicit (or explicit) learning of the rule, no difference between conditions would emerge. Additionally, as recent studies point to earlier deviance responses than the MMN (Escera et al., 2014), we used an analysis strategy that did not make any assumptions regarding the temporal specificity of predictability effects.

## Materials and Methods

### Participants

Twenty-seven adults (14 female, mean age 25 ± 4 years, ranging from 18 to 35) participated in this experiment. All participants were free from neurological or psychiatric disorder, and reported normal hearing. One participant had professional musical education and has been excluded from the analysis for he did not respect the instruction to ignore the sounds. All participants gave written informed consent and were paid for their participation. Ethical approval was obtained from the appropriate regional ethics committee on Human Research (CPP Sud-Est IV – 2010-A00301-38).

### Stimuli and Sound Sequences

The large use of frequency deviance in MMN studies encouraged us to choose this acoustic feature to test the prediction error model of the MMN. However, undesirable adaptation effects are of particular importance in this particular case because of the tonotopic organization of the auditory pathways. They would in particular impact the amplitude of exogenous event-related potentials (ERPs) in the P50 and N1 wave latency range. We therefore introduced a supplementary condition in order to control for such adaptation effects, using intensity deviance (see below). Overall, three kinds of sequences were used: (1) an unpredictable sequence with frequency deviance: UF, (2) a predictable sequence with frequency deviance: PF, and (3) an unpredictable sequence with intensity deviance: UI. Note that we did not considered a predictable sequence with intensity deviance for the sake of experiment length and also because the feature specificity of the prediction error model of the MMN is beyond the scope of the current study. All the sequences shared the same deviant probability (*p* = 0.17).

Sound duration was 70 ms (including 5 ms rise-time and 5 ms fall-time) and the stimulus onset asynchrony (SOA) was fixed to 610 ms. Two different frequencies (*f*_1_ = 500 Hz and *f*_2_ = 550 Hz) and two different intensities (*i*_1_ = 50 dB SL (*sensation level)* and *i*_2_ = 60 dB SL) were combined to define the four different stimuli that were used across conditions. In this (passive) study, we carefully chose the deviance magnitude in the frequency sequences in order to satisfy a trade-off between eliciting a deviance response, on the one hand, and both minimizing refractoriness effects and avoiding to attract the subject’s attention, on the other hand. Therefore, although even smaller deviance have been previously used (Sams et al., 1985), we used a 10% deviance which falls in the lower range of recently implemented deviance magnitudes [e.g., 8% in (Daikhin and Ahissar, 2012), 10% in (Schwartze et al., 2013), 23% in (Recasens et al., 2014), 30% in (Grimm et al., 2011), and 50% in (Todd et al., 2014)].

**FIGURE 1:**
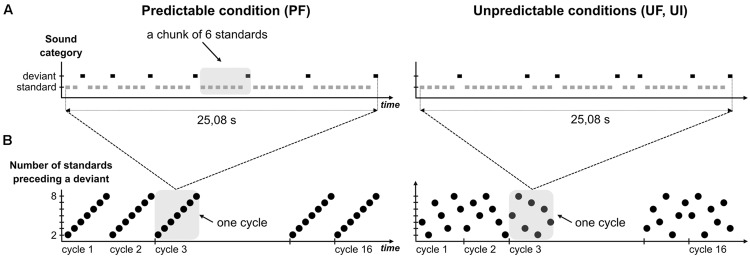
**Experimental design. (A)** Schematic view of a complete cycle in predictable (left) and unpredictable conditions (right). Rectangles symbolize single tones with standards and deviants colored in gray and black, respectively. Sound duration was 70 ms with stimulus onset asynchrony (SOA) set to 610 ms. In every condition, each cycle entails seven deviants, each of them being preceded by a number of standards ranging from 2 to 8. A chunk of *n* standards corresponds to n+1 tones (*n* consecutive standards and the following deviant), as illustrated by the shaded area in condition PF. Chunks are sorted by their size in predictable condition, whereas these are shuﬄed in unpredictable ones. **(B)** Variation of the size of chunks (black circle) within cycles, over sound sequence in predictable (left) and unpredictable conditions (right). Each sequence is composed of 16 cycles and examples of shuﬄed cycles are presented for unpredictable conditions. Shaded areas delineating one cycle in both sequence types highlight their difference with respect to sound predictability.

To design the predictable sequences (**Figure 1**), we did not use a fixed number of standards between two deviants as in Scherg et al. (1989), because this cannot be mirrored in the unpredictable sequence without inducing different refractoriness effects. This issue could be avoided by the construction of a statistical structure unfolding over a larger time-scale. Precisely, the rule that we designed increments the number of standards progressively within a cycle: it starts with one deviant after two standards, followed by one deviant after three standards and so on until one deviant after eight standards. From now on, a chunk with *n* standards will refer to a series of *n* standard sounds ending with a deviant stimulus (*n* ranging from 2 to 8). The 42-tone cycle, composed of seven incrementing chunks, was repeated 16 times in the sequence, thus leading to a total of 560 standards and 112 deviants. For the unpredictable sequences, each cycle was shuﬄed so as to permute the order of the seven chunks with the constraint that no chunk with *n* standard was preceded or followed by a chunk with either *n-1* or *n+1* standards. Additionally, the transition between two cycles was such that no successive chunks with *n* standards could occur. Altogether this randomization allowed to (1) avoid any global rule to emerge in the unpredictable sequence and (2) have exactly the same number of chunks with *n* standards in predictable and unpredictable conditions. Note that the number of deviants presented at a 2–3 chunk timescale may differ between UF and PF (for instance, the set of 16 sounds that precede a “chunk of 8 standards” deviant comprises exactly one deviant in PF and two deviants on average in UF) but the fact that adaptation saturates rapidly [2–3 standard repetitions, (Demarquay et al., 2011)] led us to assume that this particularity did not introduce any significant adaptation effect difference between PF and UF, in the current analysis that we conducted with standards just preceding deviants.

Each sequence type (UF, PF, UI) was delivered twice in separate blocks resulting in 224 deviants in each condition. For each type of deviance (frequency or intensity), the sound property used as the standard (e.g., for frequency deviance, f_1_) for the first block was used as the deviant for the second (reverse) block. The irrelevant feature was constant within a block but changed between the two reverse blocks [e.g., for frequency deviance, first block with properties (f_1_,i_1_) for standards and (f_2_,i_1_) for deviants, and reverse block with properties (f_2_,i_2_) for standards and (f_1_,i_2_) for deviants]. The order of the six resulting blocks was counterbalanced between participants with the constraint that no successive sound sequences of the same kind could be delivered. Additionally, in order to avoid any bias of perceptive association between frequency and intensity, half of the participants received the associated properties (f_1_,i_1_) and (f_2_,i_2_) as standards whereas the other half received the pairs (f_1_,i_2_) and (f_1_,i_2_). Altogether these acoustical matching constraints on stimuli and sequences were applied to ensure comparisons between conditions with an optimal control for undesirable effects of specific acoustic properties.

All stimuli were delivered using Presentation software (Neurobehavioral Systems, Albany, CA, USA).

### Procedure

The present study was conducted using simultaneous EEG and MEG recordings, although the MEG data will not be analyzed here. Participants were seated upright in a comfortable armchair in a sound-attenuated, magnetically shielded recording room, at a 1 m distance from the screen. Sounds were presented binaurally through air-conducting tubes using Etymotic ER-3A foam earplugs (Etymotic Research, Inc., USA). Participants were instructed to ignore the sounds and watch a silent movie of their choice with subtitles. Before recordings, participants’ sound detection thresholds using the sound with (f_1_,i_1_) characteristics were determined for each ear, and the level was adjusted so that the sounds were presented at 50 dB SL (i_1_) or 60 dB SL (i_2_) with a central position (stereo) with respect to the participant’s head. Each of the six blocks lasted 7 min resulting in a total recording time of ∼50 min, including short breaks between sequences. At the end of the experiment, participants were asked to report to which extent they had been following the instruction to ignore the sounds and whether they had noticed the different sound attributes (e.g., “*Did you notice anything in particular about the sounds?*”) and sequence temporal regularities (e.g., “*Did you notice that some sounds were less frequent than others?”, “Did you notice any regularities in sound presentation?*”).

### EEG Recordings

EEG recordings were carried simultaneously to MEG ones using the EEG recording system provided with the MEG equipment (275-channel whole head system, CTF-275 by VSM Medtech Inc.). EEG data were collected from 63 electrodes (including the two mastoids) whose locations were defined by the 10–15 extension of the international 10–20 system. Reference electrode and ground electrode were placed on the tip of the nose and left shoulder, respectively. One bipolar EOG derivation was recorded from two electrodes placed on the supra-orbital and infra-orbital ridges of the left eye. Throughout the recordings, impedances were below 15 kΩ. Signal was amplified, band-pass filtered (0.016–150 Hz), digitized (sampling frequency 600 Hz) and stored for off-line analysis. Head position relative to the MEG sensors was acquired continuously (continuous sampling at a rate of 150 Hz) using coils placed at three fiducial points (nasion, left and right preauricular points).

### Data Preprocessing

The software package for electrophysiological analysis (ELAN^1^) developed at the Lyon Neuroscience Research Center (Aguera et al., 2011) was used for ERP computation and statistical analysis.

EEG and MEG data were preprocessed independently but for the sake of a combined analysis, which will be reported in a further study, we only used time epochs that survived the procedures applied for artifact rejection for both techniques. A total of 5 participants out of 27 had to be excluded from the group. For two participants, raw MEG recordings were contaminated by ferromagnetic artifacts caused by metallic elements, which created a temporally stationary artifact at the participant’s respiratory frequency. One participant’s EEG data had a very bad SNR. One participant had individual MR images that disclosed a ventriculomegaly. Finally, as mentioned above, one participant did not ignore the sounds as instructed but counted them leading to an explicit detection of the predictable rule in PF sequences. Preprocessing of raw data for the remaining 22 participants comprised the following successive steps: (1) an initial rejection of data segments corrupted by head movements above 15 mm within each sequence was automatically performed (in prevision of future MEG data analysis), (2) three stop-band filters centered on 50, 100, and 150 Hz (with bandwidth of ±2 Hz) were applied to get rid of the power line artifact in the EEG data, (3) using EEGlab routines^2^, an independent component analysis (ICA) correction for ocular artifacts was achieved (largest possible time windows – free from artifacts from all origin but ocular – were selected from continuous stop-band filtered data to derive ICA components) for all participants but one for whom ICA correction failed to improve the SNR of EEG and MEG data, (4) individual recordings were automatically inspected from -200 ms to 410 ms with respect to the onset of each sound; trials with signal amplitude range exceeding 2000 fT for MEG data and 150 μV for EEG data over the 610 ms time-window at any sensor were excluded from the analysis (for the participant whose data did not receive any ICA correction, a threshold of 100 μV was used for the EOG signal range), (5) a 2–45 Hz band-pass digital filter (bidirectional Butterworth, fourth order) was applied to EEG and MEG data. It should be noted here that most MMN studies rely on filtered data with lowpass cutoff frequency lower than 45 Hz (20 or 30 Hz are commonly used), leading to *smoother* baselines and ERPs.

### Event-Related Potential (ERP) Computation

Data collected within the first 20 s of each block was excluded from averaging to ensure that no transitory effect could bias the ERPs. Responses to standards just preceding a deviant and to deviants were considered for averaging within an epoch of 610 ms including a pre-stimulus period of 200 ms. Baseline correction was achieved by subtracting the mean value of the signal during the pre-stimulus period. ERPs for each stimulus type (standard and deviant) were first computed per block. The two reverse blocks for each condition (UF, PF, and UI) were then pooled by averaging corresponding ERPs. Difference response (also referred to as deviance response) was obtained by subtracting the standard ERP from the deviant one.

### Statistical Analysis

We applied permutation tests based on a *t*-statistic at the group-level at each sample of each electrode of the ERP time series in bandwidth 2–45 Hz, correcting for multiple comparison in the temporal dimension (Blair and Karniski, 1993; Besle et al., 2008). For each test, we ran 100,000 permutations by randomly redistributing the ERPs of the two conditions to be compared. We tested for (1) an effect of deviance in the three conditions (i.e., standard vs. deviant in UF, UI, and PF), (2) an effect of predictability (i.e., PF vs. UF) in difference, deviant and standard responses, (3) an effect of acoustic features (i.e., UF vs. UI) in the difference, deviant, and standard responses. Finally, since the first analysis above revealed a significant effect of deviance at both early and late latencies as well as a smaller effect at the P3a latency, we also conducted further analysis in tests (2) and (3) in three local time windows [0, 80] ms, [100, 210] ms and [250, 350] ms. Hence, permutation tests were run both on the entire time series [-200, 410] ms for each effect of interest (1, 2, 3) and on specific local time windows for (2, 3).

### Adaptation Effect Characterization

To isolate the effect of predictability on genuine mismatch responses in conditions UF and PF, we had to characterize the effect of adaptation. Our experiment was designed to minimize this effect and we hypothesized that, if present, it would be the same in the UF and PF conditions. To this aim, we used a small deviance magnitude to reduce refractoriness effect as much as possible and imposed strong acoustical constraints on sound sequences such as a strict balancing of the number of standards preceding a deviant across conditions. Moreover, we introduced a third condition using an intensity deviance (condition UI) as a control condition for these possible adaptation effects. Adaptation effects for intensity deviance cannot be ruled out, although their existence remains rather controversial [but see Bilecen et al. (2002)]. We assumed that the MMN to intensity would not be contaminated by refractoriness, or at least to a far smaller extent than the MMN to frequency. Furthermore, we carefully matched the intensity and frequency deviance magnitude thanks to a prior behavioral deviance detection task so that frequency and intensity MMN would have similar amplitudes. Consequently, comparison between UI and UF *difference* responses should help characterizing (in the temporal and spatial dimensions) the undesired adaptation effects possibly entering UF and PF *difference* responses.

### Control for Possible Filtering Confounds in Early Effects

As early effects were revealed by statistical tests in both the deviant vs. standard and the predictable vs. unpredictable comparisons, additional analysis were needed to control for their validity. As explained in Acunzo et al. (2012), the bidirectional low-pass filter that we applied on our data may have generated artifactual responses preceding the sharper deflections of the ERPs, namely the N1 and MMN components. In order to test whether our early effects were of such artifactual origin, we repeated the whole ERP analysis (using the statistical analysis described above) on unfiltered data to control for any bias induced by filtering (particularly low-pass filtering). These unfiltered data correspond to the data recorded by the acquisition system (0.016–150 Hz acquisition bandwidth) with further application of three stop-band filters and ICA correction as described in the Data processing section. Trials averaged for both ERP types (standard and deviant) were those retained for the analysis in the 2–45 Hz bandwidth. Note that this complementary analysis also allows to check that the 2 Hz high-pass filter that we used for the main analysis did not obscure some differences between conditions, e.g., in the very low frequencies.

## Results

Post-experimental debriefing with the 22 participants whose data were retained for statistical analysis (11 female, mean age: 25 ± 5 years, ranging from 18 to 35) revealed that 15 of them noticed that sounds could take different intensities, 12 noticed that sounds could take different frequencies and nine noticed that some sounds were less frequent than others. Critically, none of them reported to have inferred the global rule of the PF sequence. Given our design, this implies that any difference between deviance responses in UF and PF reflects implicit learning of a global rule in PF.

On average per subject, the number of retained standard trials (standard sounds just preceding a deviant sound) was 177 ± 16 for the UF sequence, 174 ± 18 for the UI sequence and 172 ± 17 for the PF sequence. Similarly for deviants, the number of retained trials was 174 ± 17 for the UF sequence, 172 ± 22 for the UI sequence and 172 ± 21 for the PF sequence.

### Multiple Deviance-Specific Responses

**FIGURE 2:**
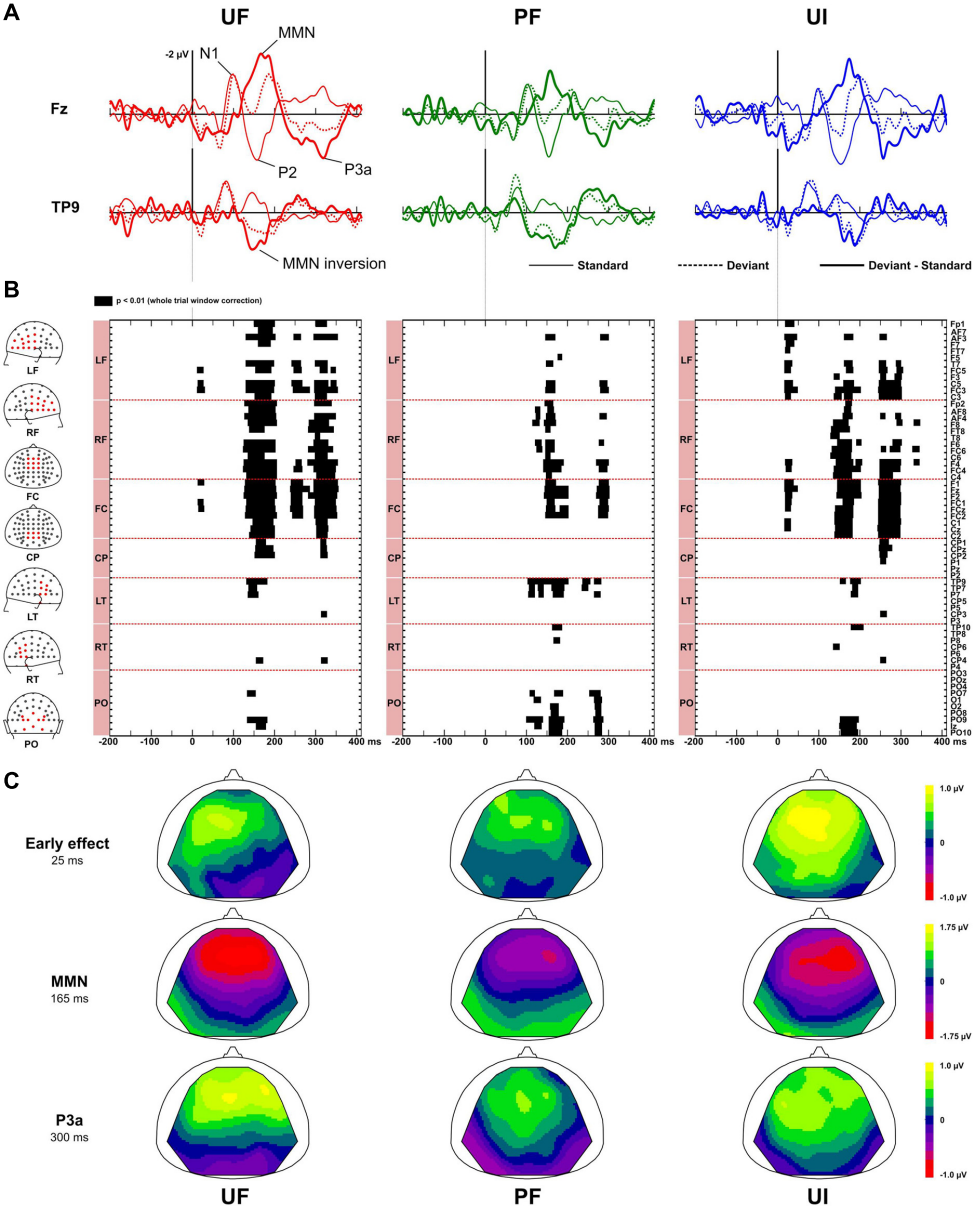
**Deviance effects. (A)** Grand-average ERPs (*n* = 22 participants) elicited by standards just preceding a deviant (solid line), deviants (dotted line) and difference responses (bold solid line) at electrode Fz and TP9 in bandwidth 2–45 Hz for condition UF (left column), PF (middle column), and UI (right column). Main components in standard (N1, P2) and difference responses [mismatch negativity (MMN), P3a] are shown for condition UF. **(B)** Statistical maps obtained with non-parametric tests (*n* = 100,000 permutations) when comparing standard and deviant responses, at each electrode and each latency of the whole trial. Three intervals of significance were revealed for unpredictable sequences (UF, UI) at early latencies, and at the latency of the MMN and the P3a whereas only two were observed for condition PF at the latency of the MMN and P3a. Electrodes are sorted by spatial clusters (left column, from top to bottom: LF, left frontal, RF, right frontal, FC, fronto-central, CP, centro-parietal, LT, left temporal, RT, right temporal, PO, parieto-occipital). **(C)** Scalp topographies of the grand-average difference ERP, at the early effect (left column), the MMN (middle column) and the P3a (right column) latencies, for each condition. The MMN significant (positive) inversion is visible in each condition. Similarly, early deviance effect in condition UF and UI also entail a (negative) inversion but this does not reach significance.

**Figure 2** displays ERPs (with bandwidth 2–45 Hz) at electrodes Fz and TP9, for the standard, deviant, and difference responses, in each experimental condition. It also shows the statistically significant patterns in the deviance responses and the corresponding scalp topographies at relevant latencies. In every condition, the standards just preceding a deviant elicited a N1 component peaking around 95 ms, associated with a negativity distributed over fronto-central electrodes and followed by a fronto-central P2 component peaking around 155 ms. As shown on **Figure 2**, testing for deviance effects revealed three significant time-windows for the unpredictable sequences and two for the predictable one: an early time-window (within 70 ms after stimulus onset) for conditions UF and UI, and for the three conditions, we could detect a MMN and a P3a.

**FIGURE 3:**
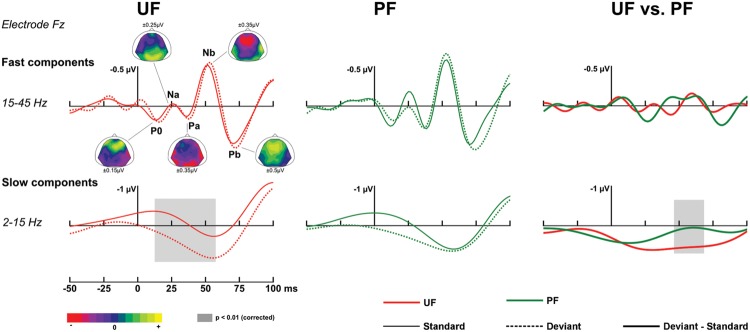
**Early responses.** Traces at electrode Fz in the time interval [-50, 100] ms, with original [-200, 0] ms baseline correction. ***Fast components, bandwidth 15–45 Hz*** (top row). Grand-average ERPs elicited by standards just preceding a deviant (solid line) and deviants (dotted line) for condition UF (left column) and PF (middle column). Data were re-referenced to the average of both mastoids to facilitate the identification of Middle Latency Responses (MLR) components. Fast MLR components are indicated for condition UF with corresponding scalp topographies (from standard ERPs, with original nose reference allowing for the visibility of temporal polarity inversion) at the latencies 13, 26, 36, 50, and 68 ms for P0, Na, Pa, Nb, and Pb, respectively. Right column: Grand-average ERPs corresponding to difference responses (bold lines) for condition UF and PF. ***Slow components, bandwidth 2–15 Hz*** (bottom row).

*At early latencies*, larger responses were elicited with deviants in condition UF compared to standards, leading to a positive difference response spanning from about 10 to 90 ms over the frontal and central areas. It was confirmed statistically significant from 11 to 28 ms at six adjacent electrodes located in left fronto-central area (-0.2 and 0.6 μV at Fz at 20 ms for standards and deviants, respectively). In condition UI, the deviant response was very similar to the one in UF, thus leading to very similar difference responses (deviant – standard) in those two conditions. Statistical analysis for UI revealed a significant interval occurring from 16 to 38 ms on left frontal and fronto-central areas. On the contrary, in condition PF, no significant effect was found at this early latency range. Because at this early latency there is an overlap of slow components (such as the P50) and fast Middle Latency Responses (MLR), we ran a complementary analysis with two different filtering (2–15 and 15–45 Hz) to further characterize this deviance effect. As shown on **Figure 3**, statistical analysis in the bandwidth 2–15 Hz confirmed the significant early deviance effect measured in UF (from 13 to 58 ms) whereas statistical tests in the bandwidth 15–45 Hz did not reveal any significant effect. A similar pattern was observed for condition UI (data not shown). Altogether, these results suggest that early deviance effects measured here in UF and UI pertain to a slow component at the latency of the P50 and do not concern the peaks of the MLR *per se*.

*In the MMN latency range*, difference response in condition UF showed a typical MMN peaking around 165 ms, with large negativity over the frontal electrodes (-1.9 μV at Fz) combined with a positivity at the mastoids (the MMN inversion), with both deflections ending at the same latency. A similar difference response was observed in condition UI. The emergence of the MMN was statistically significant from 125 to 205 ms over 33 fronto-central electrodes and mastoids for UF, and from 128 to 205 ms over fronto-central electrodes, mastoids and occipital electrodes for UI. In condition PF, the difference response revealed the MMN inversion starting around 100 ms over the parieto-occipital areas, followed by the MMN *per se* (-1.4 μV at Fz), peaking at about 156 ms with a large negativity over frontal electrodes. Statistical tests confirmed the emergence of the MMN inversion (from 105 to 200 ms over mastoid and occipital electrodes) and of the MMN proper (from 120 to 200 ms over fronto-central electrodes and parieto-occipital electrodes). In all three conditions, the MMN inversion ended at the same latency than the frontal negativity deflection, suggesting that the N2b component, which does not invert in polarity at the mastoids, was negligible if any.

*Finally, in the P3a latency range*, a large positive deflection at fronto-central electrodes could be seen for difference responses of all conditions. These typical P3a components were maximal at around 316, 295, and 290 ms for UF, UI, and PF, respectively (with corresponding peak amplitude at Fz: 1.4, 0.8, and 1.0 μV for UF, UI, and PF, respectively). For condition UF, the emergence of the P3a was statistically significant from 238 to 270 ms over 12 frontal and fronto-central electrodes, and from 295 to 355 ms over 31 fronto-central and centro-parietal electrodes (including Fz, FCz, Cz, and CPz). Similarly, for condition UI, emergence was significant from 245 to 303 ms over 26 fronto-central and centro-parietal electrodes (including Fz, FCz, Cz, and CPz). For condition PF, statistical significance was measured from 265 to 281 ms over nine temporal and parieto-occipital electrodes (including TP9, P0z, and Iz), and from 280 to 303 ms over 13 frontal and fronto-central electrodes.

### Predictability Modulates the Early Deviance Response, the MMN and the P3a

**FIGURE 4:**
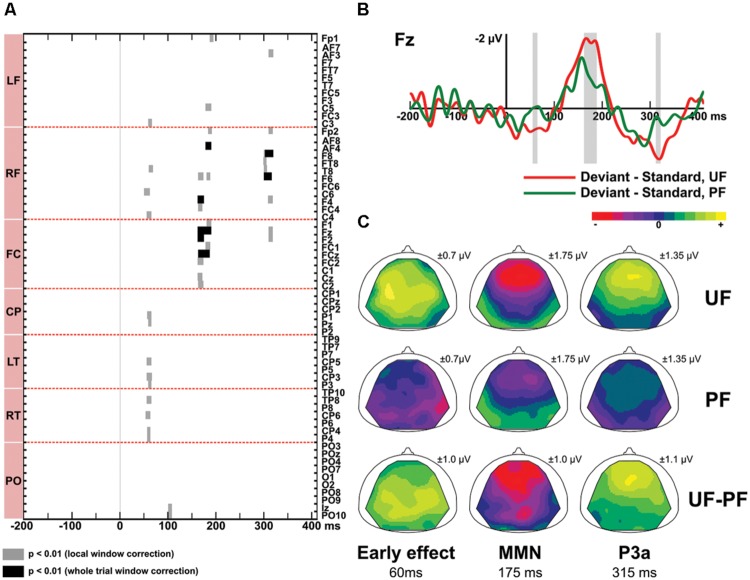
**Predictability effect (UF vs. PF). (A)** Statistical maps of the permutation tests comparing difference responses between condition UF and PF, at each electrode and each latency of the whole trial. Black and gray areas indicate significant differences (*p* < 0.01) resulting from whole trial [-200, 410] ms and local tests, respectively. Results revealed three intervals of significant difference: at early latencies (13 electrodes), at the latency of the MMN (15 electrodes) and at the latency of the P3a (7 electrodes). **(B)** Grand-average ERPs elicited by difference responses at electrode Fz in bandwidth 2–45 Hz for condition UF (red) and PF (green). Shaded areas display the windows of statistical significance (at any electrode). **(C)** Scalp topographies of the difference responses in bandwidth 2–45 Hz, at the latency of the predictability effect, for the early effect (left column), the MMN (middle column) and the P3a (right column), in conditions UF and PF. The range of voltage values used for the color scale is mentioned for each map.

**Figure 4** displays difference responses for conditions UF and PF at electrode Fz, as well as scalp topographies of the double difference waveforms (UF difference response – PF difference response). The effect of predictability was first assessed by comparing the difference responses obtained with the predictable and unpredictable sequences (PF vs. UF). Second, in order to disentangle the relative contribution of standard and deviant stimuli, we further assessed the effect of predictability on those two responses, separately.

*Difference responses* (**Figure 4**) differ as early as around 35 ms due to a weak (non-significant) deviance response measured in PF whereas a large significant fronto-central positivity was measured in UF (see above). It was confirmed significant from 55 to 65 ms on 13 electrodes, with more positive potentials in UF compared to PF (at 60 ms, 0.6 and -0.04 μV at Fz for UF and PF, respectively). Moreover, statistical analysis in the bandwidth 2–15 Hz revealed a significant effect from 46 to 68 ms (14 electrodes). No significant effect was found in the 15–45 Hz frequency band (**Figure 3**). Following this early effect, the scalp topography of the double difference (**Figure 4**) shows that the MMN peak is larger in the UF condition than in the PF one (from 163 to 190 ms over 15 fronto-central electrodes). We also observed a tendency for the MMN inversion in the PF condition to start earlier than in the UF condition (from about 100 to 130 ms) and to be enhanced at parieto-occipital electrodes from about 150 to 210 ms, but these effects were not statistically significant. Finally, the statistical analysis also revealed a larger P3a component in UF compared to PF (at 315 ms, 1,4 and 0.3 μV for UF and PF, respectively at Fz), with significance spanning from 310 to 320 ms over seven electrodes (Fp2, AF3, Fz, F2, F4, F6, F8).

*In response to deviants*, permutation tests confirmed that more positive potentials were recorded in UF compared to PF in the early latency range (at 65 ms, 1.1 and 0.7 μV for UF and PF, respectively at Fz), with significance spanning from 58 to 72 ms over 18 fronto-central and left centro-parietal electrodes. Moreover, the negative deflection following the N1 was significantly larger in UF from 178 to 190 ms at electrodes F1, F3, Fz, FC1, FCz, and FC3 (at Fz: -1.2 μV at 185 ms for UF, and -0.8 μV at 205 ms for PF). These two effects observed for the deviant thus mirrored those observed in the difference wave tests. At the latency of the P3a, no significant difference between UF and PF could be measured.

*In response to standards*, no significant effect of sequence predictability could be observed in the ERPs of standards just preceding a deviant. Larger N1 and P2 components were observed in the UF compared to the PF condition (see **Figure 2**, standard traces at electrode Fz for UF and PF) but this tendency did not reach significance.

To sum up, an effect of predictability was observed, not only at the latency of the MMN but also earlier, within 70 ms after deviant onset. These two effects go as expected: the more predictable the sequence, the smaller the deviance response. The P3a component was also modulated by the sequence predictability, with larger amplitude observed in UF. The first two effects seem to derive mostly from a deviant response contribution, the P3a one could not be statistically attributed to either standard or deviant responses only.

### Controls for Non–Predictability based Biases in UF and PF Responses

First, characterization of undesirable adaptation effects in frequency deviance sequences (UF and PF) was achieved by the comparison between UF and UI conditions. Statistical tests did not reveal any significant effect neither on the difference response (with the exception of TP9 and TP7, from 136 to 148 ms), nor on the deviant and standard responses taken separately, suggesting that the deviance effects observed in UF are, at least to a large extent, not resulting from undesirable refractoriness effects on exogenous ERPs (P50, N1 in particular).

Second, statistical analysis of unfiltered ERPs confirmed every significant effect reported above in bandwidth 2–45 Hz. However, it should be noted that the spatial and temporal extents of those effects were reduced with unfiltered data, which is perfectly sensible at lower SNR. In the Supplementary material, we provide the unfiltered difference responses for conditions UF and PF at electrode Fz, as well as the corresponding statistical maps obtained from the permutation tests.

## Discussion

In this study, we measured different deviance responses elicited by oddball sequences only differing by their statistical temporal structure, referred to as predictability. Our results indicate that sequence predictability modulates deviance responses such that the more predictable the deviant stimulus, the smaller the deviance response. This modulation affects not only the MMN but also earlier slow responses, at the latency of P50 and the auditory MLR components, thereby arguing in favor of various mismatch responses reflecting prediction errors and updates at different levels of the auditory hierarchy. In addition, the measured modulation of the P3a is consistent with unpredictable deviants inducing a larger attentional capture effect. Importantly, these effects were elicited while participants were unaware of the sequence structure. This substantiates the ability of the brain to implicitly monitor statistical properties of the environment such as sequence predictability.

### Deviance Effects are not Confounded with Adaptation Effects

Regarding deviance responses, refractoriness state difference between UF and PF should be minimized by the sequence design, which involves the same number of stimulus chunks of each size for both conditions. Moreover, UI and UF deviance responses did not significantly differ, suggesting that not only these responses are similar for both features but also, and more importantly, that frequency deviance of a small magnitude (50 Hz) did not elicit any refractoriness effect detectable in the EEG with our analysis strategy. These findings ensure that observed significant differences between deviant and standard responses are genuine deviance effects. We can thus assume that the significant difference between deviance responses observed in condition UF and PF is not confounded with adaptation effects.

### Sequence Predictability Reduces MMN Amplitude

Contrary to Scherg et al. (1989), we measured a significant modulation of the MMN amplitude by sequence predictability, which we interpret as reflecting a smaller prediction error due to a more predictable deviance occurrence. In Scherg et al. (1989), the absence of effect has been interpreted as a result of the automaticity of the MMN, which would prevent this component from being modulated by high-level cognitive processes such as rule extraction. It should be noted that their result derived from a preliminary study conducted with only five participants and relied on a statistical analysis focusing on the MMN amplitude at electrode Fz. Visual inspection of deviance responses for a deviance magnitude of 50 Hz (see Figure 3 in Scherg et al., 1989) shows a difference between regular and irregular sequences which is compatible with our findings. It then appears plausible that a more comprehensive analysis, over all sensors and time bins, would reveal a significant modulation by predictability. However, their experimental design was not adapted to characterize the effect of predictability in isolation from any possible refractoriness confound.

The reduction of the MMN amplitude when predictability of deviance occurrence increases is in line with predictive coding or the Bayesian brain hypothesis (Knill and Pouget, 2004; Friston, 2005). It allows formulating interpretations regarding the underlying mechanisms of prediction updating. UF and PF sequences only differ by their statistical regularities (brought by the global rule). In condition PF, exposure to at least two or three incrementing chunks is required in order to start inferring the regularity of the sequence; with the more chunks, the stronger the confidence in that rule. Perceptual learning - here defined as the process by which the brain encodes over trials the statistical structure of a sensory environment (Friston and Stephan, 2007) -by contrast with the process of learning of new perceptual skills [like in Alain et al. (2007) for instance]- could thus explain the observed modulation of the MMN in the PF compared to UF condition. Predictions, which are updated dynamically through sequential exposure to the stimuli, could indeed be refined in PF through the learning, although approximate, of sequence statistical dependencies. Importantly, none of the participants did report being aware of the differences between experimental conditions. As instructed, they obviously paid little attention to the sounds. This interpretation is consistent with the small amplitude measured for the N2b and P3a components, as we know that they typically follow the MMN under specific condition of stimulus salience or attention orienting toward the stimulus. Altogether, these findings strongly suggest that those perceptual learning processes are implicit. A large number of studies have proposed that the MMN elicited by the violation of complex rules indirectly evidence the implicit learning capacities of the brain. Beside oddball paradigms, the brain ability to track and learn abstract rules without awareness has been straightforwardly evidenced by a large number of studies in the fields of implicit and statistical learning (Perruchet and Pacton, 2006). In line with these accounts, our data argue for a unified implicit learning process that optimizes predictions at different levels. Hence the brain would be constantly tracking the regularities of the environment by means of statistical and implicit learning so as to infer the hidden causal rule(s) governing incoming sensations. Throughout this inference process, mismatch responses would reflect the dynamics of prediction updating, which is guided by the minimization of prediction errors (Friston, 2005). The decrease of mismatch responses observed for the predictable sequence gives support to the idea that the brain optimizes its predictions, even independently of awareness. The MMN has already been proposed to be weighted by the confidence about predictions established through stimulus exposure (Winkler et al., 2009; Todd et al., 2014). Interestingly, the presence of an MMN in condition PF suggests that prediction errors were not abolished for the fully predictable sequence. This could be due to the predictions derived from the approximate learning of the global rule but also to the fact that the local (repetition) rule in UF is still valid in PF sequences. Despite the existence of high-level predictions derived from the learned global rule, low-level predictions integrating incoming information on a short time-scale might still generate prediction error signals. This is in line with Horváth et al. (2001) who demonstrated the simultaneous integration of different rules at different time-scales, and with Kiebel et al. (2009) pointing to different time-scale prediction errors, corresponding to different levels of an internal hierarchical model.

Under the predictive coding view of the MMN, one could expect the predictability effect to affect both responses to deviants and standards. However, for the latter we only observed a tendency of smaller N1 and P2 responses to predictable standards but no statistically reliable difference. One possible explanation for this lack of significance relates to the passive nature of this paradigm that induces rather small responses to standards, thus yielding a poor signal-to-noise ratio when comparing PF and UF.

Note that in the current study, we manipulated simple perceptual stimuli and observed a modulation of automatic sensory processes by temporal predictability. It would be interesting to replicate our paradigm with conceptual stimuli to test whether this contextual modulation also operates on higher-level processes. Our prediction is that the same effects would be observed and likely express on later components related to more abstract processes like those pertaining to semantic information for instance.

### Early Markers of Deviance Detection and Deviance Predictability

Contrary to the majority of MMN studies, we conducted our statistical analysis on entire epochs (from -200 to 400 ms) and this strategy revealed earlier markers of mismatch than the MMN for the unpredictable sequences (UF, UI), within 70 ms after deviant onset. We could identify a statistically significant deviance effect at low frequencies (below 15 Hz). It is worth noting that our set-up and experimental design was not adapted for a fine characterization of fast MLR components, which can also be modulated in oddball paradigm (see below), as there were only ∼175 trials retained on average per stimulus type (typically over 1000 for MLR studies), and an upper bound of bandwidth limited to 45 Hz (typically 150 Hz or 200 Hz for MLR studies). Critically, the genuineness of these early responses had to be controlled with regard to adaptation effects and high-pass filtering bias. Results of these tests, namely an absence of significant difference between UF and UI responses and all effects measured in the bandwidth 2–45 Hz retrieved significantly with unfiltered data, allow us to conclude with high confidence in favor of *genuine* deviance responses for every early effect reported in this study.

Recent findings have already confirmed deviance processing within 50 ms after stimulus onset (for review see Grimm and Escera, 2012; Escera et al., 2014). Contrary to the current results, these findings pertain to the rapid components of the MLR with for instance, an enhancement of the Nb component elicited with pure tone frequency deviants measured with EEG (Grimm et al., 2011) and MEG (Recasens et al., 2014) recordings. Such early mismatch responses complement single-neuron recordings (in animal studies) showing novelty detection responses within midbrain, thalamus and primary auditory cortex (Ulanovsky et al., 2003; Ayala and Malmierca, 2012). Interestingly, Escera and Malmierca (2014) proposed a model of the auditory system dedicated to deviance detection processing at the latency of the MLR that unifies scalp and neuron level findings. Together with the current results, these findings suggest that deviance processing expresses very early and affects both the fast and slow components of the deviant response at early latencies.

Predictable and unpredictable deviance responses were also measured significantly different from about 60 ms over temporo-parietal electrodes. As for the MMN modulation by sequence predictability, we propose that implicit learning is the key mechanism that explains how such early components can be shaped by a global rule. The predictability effect at both early and late latencies could reflect a modulation of high-level predictions on low-level ones within the deviance processing hierarchy. Besides, our results confirm sequence predictability as a suitable tool to characterize the different components of deviance response properly.

Interestingly, previous studies of early deviance effects failed to measure such early ERPs after a global rule violation (Cornella et al., 2012; Althen et al., 2013; Recasens et al., 2014). Escera et al. (2014) and Escera and Malmierca (2014) suggest that these findings corroborate the hierarchical organization of the auditory system, where the different time-scales defining the regularities of the environment would be processed in a forward direction. This model is totally in accordance with a predictive coding implementation (Kiebel et al., 2009), where early deviance responses and the MMN would reflect prediction errors and updates at different levels. However, this view cannot explain the reduced (and thus non-significant) early deviance response in PF as no global rule violation occurs in this condition: mismatch responses are elicited by local rule violation just as they are in the unpredictable sequences. Hence, perceptual learning of the global context may be a plausible explanation to account for the results in PF, with high-level predictions controlling lower level ones. Hence our study provides a new (complementary) contribution to the characterization of the hierarchical auditory system, highlighting top-down (backward) modulations within this hierarchy.

### Modulation of the P3a by Sequence Predictability

Following the MMN, the P3a is widely acknowledged as reflecting attention-orienting processes (Polich, 2007). Despite the small frequency and intensity deviance magnitudes that were used, a small but significant P3a component was observed in each of the three experimental conditions. However, its small amplitude, smaller than the MMN deflections, (see **Figure 2**), suggests that the automatic orientation toward the deviants remained rather limited. Note that since the presence of a P3a cannot be interpreted as the signature of an explicit engagement of attention [for instance, it was measured during sleep (Ruby et al., 2008) and with patients with disorders of consciousness (Morlet and Fischer, 2014)], this finding remains compatible with the absence of awareness of the sequence structure as inferred via verbal report in every participant. Interestingly, sequence predictability also induced a significant modulation of the P3a with larger responses to unpredictable deviants. This further suggests that the P3a also reflect a (third) prediction error. This is definitely in keeping with the predictive coding model of deviance processing, where unexpected stimuli trigger a cascade of prediction errors (conveyed from lower levels to higher ones) that induce in turn adjustments of predictions within each level of the hierarchy. The Dynamic Causal Modeling (DCM) study of Garrido et al. (2007) supports this view, as the authors showed that frontal-to-temporal connections become necessary to explain auditory deviance responses up to the latency of the P3a. An alternative (but compatible) interpretation is that the smaller P3a in the case of predictable deviants reflects a smaller automatic shift of attention.

## Conclusion

The recent prediction error model of the MMN yields new expectations regarding its modulations by specific experimental factors, and one of them, sequence predictability, was employed here to refine our understanding of deviance processing. Indeed, we proposed a passive auditory oddball paradigm allowing for the measurement of this effect on genuine deviance responses. We observe a decrease of deviance responses induced by sequence predictability, which directly relates these ERPs to prediction errors and thereby substantiates the predictive coding scheme. Moreover, the threefold predictability effect observed at early and late latencies gives strong support to an auditory hierarchy computing prediction errors at different levels. The statistical structure of sound sequence could be encoded implicitly, possibly through a bayesian inference and learning process implemented within the hierarchy (Kiebel et al., 2009), and large time-scale regularities could induce high-level predictions that modulate both the content and the precision of lower-level ones. These new findings thus raise questions regarding the neural implementation of the predictive coding scheme and the dynamics of deviance processing within the dedicated hierarchy. Hence, further use of our paradigm, in conjunction with generative modeling approaches (Garrido et al., 2009a; Wacongne et al., 2012; Lieder et al., 2013) as well as suitable design optimization methods to compare such models (Sanchez et al., 2014) should help shedding light onto the neurocomputational mechanisms underlying rule learning and deviance processing.

## Conflict of Interest Statement

The authors declare that the research was conducted in the absence of any commercial or financial relationships that could be construed as a potential conflict of interest.

## Footnotes

1 http://elan.lyon.inserm.fr

2 http://sccn.ucsd.edu/eeglab/index.html
