# Supplementary material for: Implicit learning of predictable sound sequences modulates human brain responses at different levels of the auditory hierarchy
Source: Front Hum Neurosci. 2015 Sep 16;9:505. doi: 10.3389/fnhum.2015.00505 (PMC4584941; doi:10.3389/fnhum.2015.00505)
Supplement: Supplementary file 1 [file Image_1.PDF]

Supplementary Materials

Measure of the predictability effect (UF vs. PF) - Unfiltered data

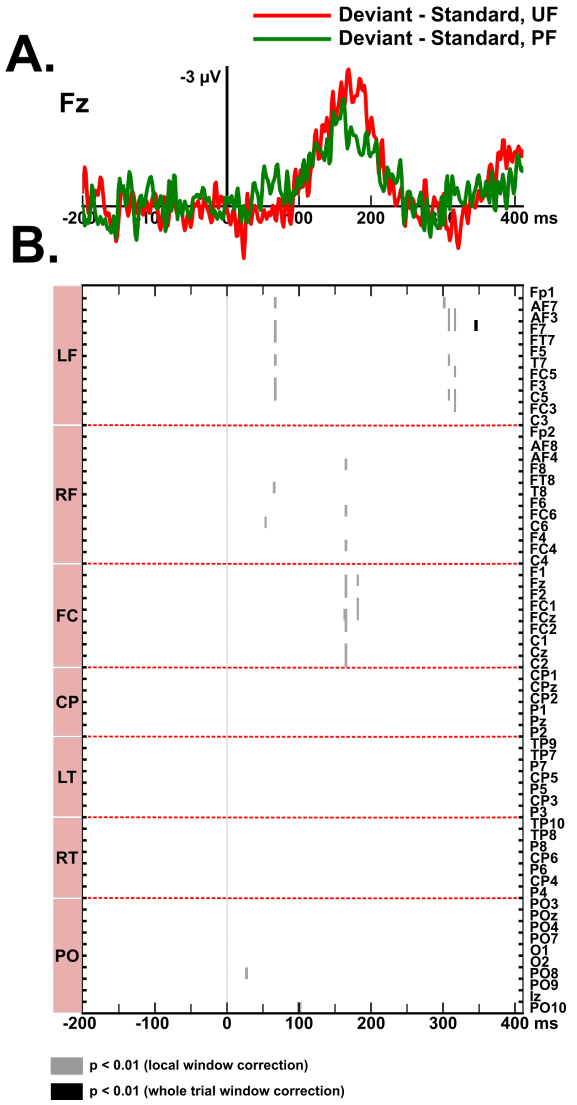

Predictability effect (UF vs. PF) with 0.016-150 Hz data.

A. Grand-average ERPs (n= 22 participants) of difference (deviant-minus-standard) responses at electrode Fz for condition UF (red) and PF (green).

B. Statistical maps of the permutation tests comparing difference responses between condition UF and PF, at each electrode and each latency of the whole trial. Black and grey areas indicate significant differences ($p < 0.01$) resulting from whole trial ([-200 410] ms) and local tests respectively. Results revealed three intervals of significant difference: at early latencies (8 electrodes), at the latency of the MMN (10 electrodes) and at the latency of the P3a (7 electrodes).
